# Supplementary figures and images for: Obesity and revision surgery, mortality, and patient-reported outcomes after primary knee replacement surgery in the National Joint Registry: A UK cohort study
Source: PLoS Med. 2021 Jul 16;18(7):e1003704. doi: 10.1371/journal.pmed.1003704 (PMC8284626; doi:10.1371/journal.pmed.1003704)

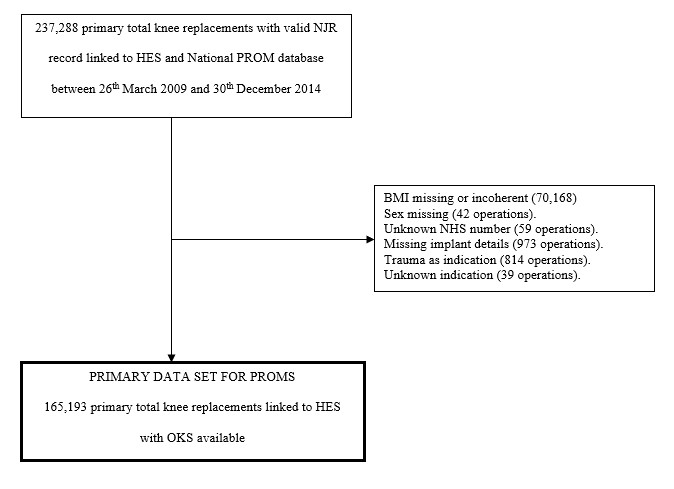

Supplement: S1 Fig — PROMs, Patient Reported Outcome Measures. (TIF) [file pmed.1003704.s002.tif]

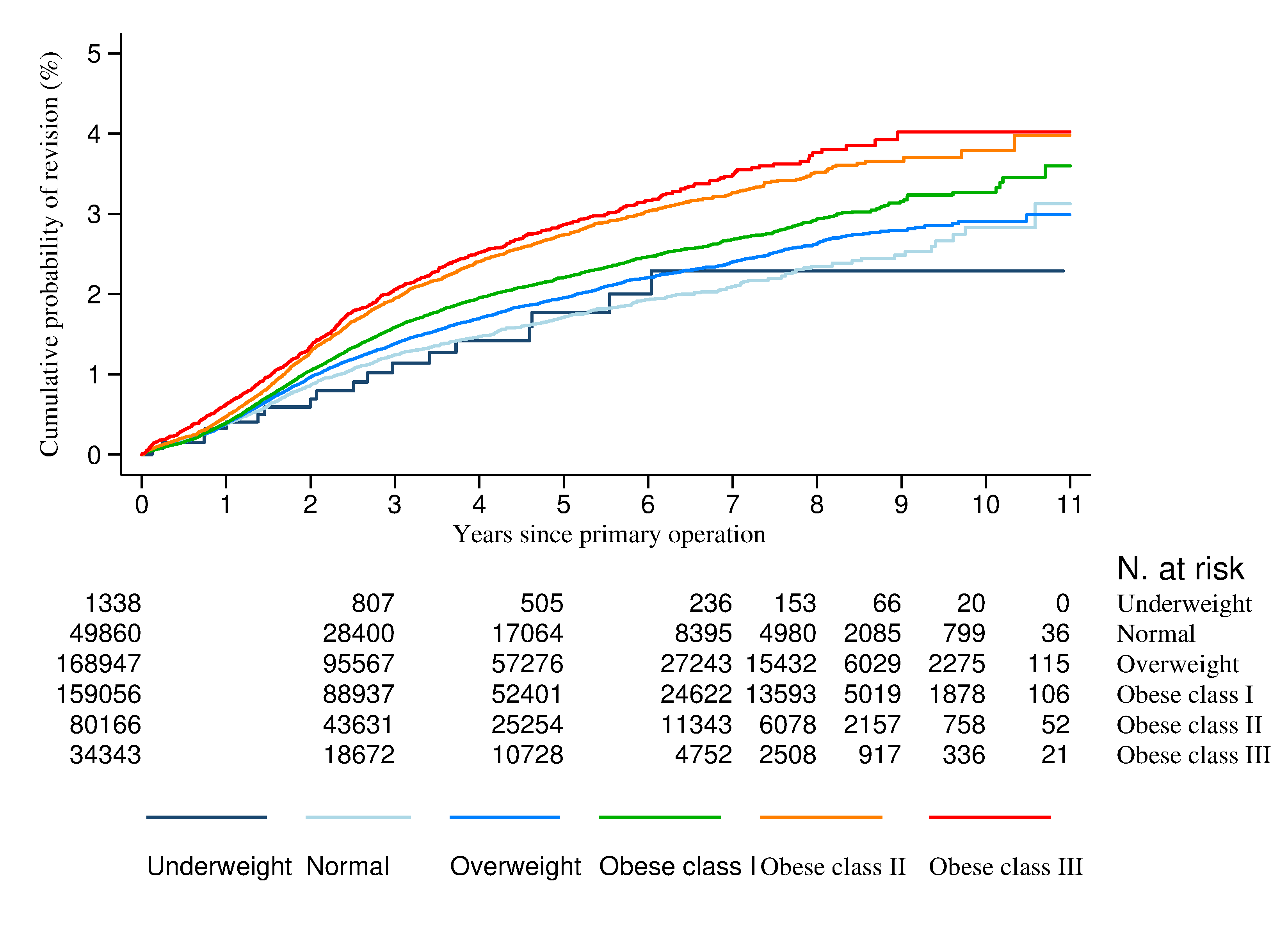

Supplement: S2 Fig — BMI, body mass index; KM, Kaplan–Meier. (TIF) [file pmed.1003704.s003.tif]

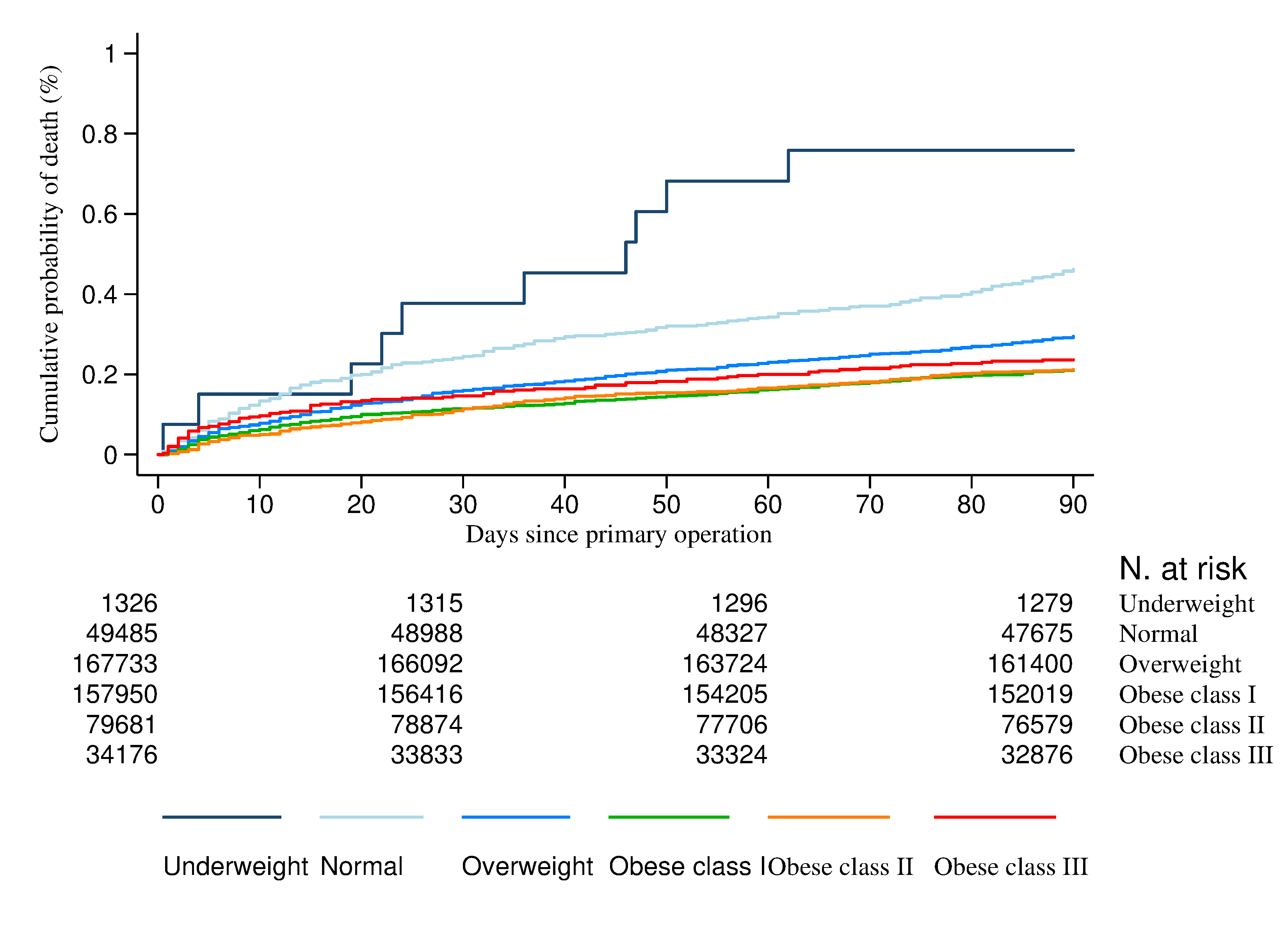

Supplement: S3 Fig — BMI, body mass index; KM, Kaplan–Meier. (TIF) [file pmed.1003704.s004.tif]
