# Supplementary material for: Obesity and revision surgery, mortality, and patient-reported outcomes after primary knee replacement surgery in the National Joint Registry: A UK cohort study
Source: PLoS Med. 2021 Jul 16;18(7):e1003704. doi: 10.1371/journal.pmed.1003704 (PMC8284626; doi:10.1371/journal.pmed.1003704)
Supplement: S1 Table — All the models were fitted on the hazard scale using 4 degrees of freedom. Adjusted models adjust for age, gender, type of fixation, ASA grade, year of having the primary operation, indication for operation, IMD, and Charlson comorbidity index. ASA, American Society of Anaesthesiologists; BMI, body mass index; HES, Hospital Episodes Statistics; IMD, Index of Multiple Deprivation; NJR, National Joint Registry; TKR, total knee replacement. (DOCX) [file pmed.1003704.s005.docx]

|  | **Unadjusted model** | | | **Adjusted model** | | |
| --- | --- | --- | --- | --- | --- | --- |
| **BMI** | **HR** | **95% CI** | **p-value** | **HR** | **95% CI** | **p-value** |
| <18·5 kg/m^2^ | 1·07 | (0·64, 1·78) | 0·805 | 0·98 | (0·58, 1·63) | 0·925 |
| 18·5–24·99 kg/m^2^ (reference) | 1·00 |  |  | 1·00 |  |  |
| 25–29·99 kg/m^2^ | 1·11 | (1·00, 1·22) | 0·047 | 1·03 | (0·93, 1·14) | 0·539 |
| 30–34·99 kg/m^2^ | 1·27 | (1·15, 1·40) | <0·001 | 1·08 | (0·97, 1·19) | 0·155 |
| 35–39·99 kg/m^2^ | 1·56 | (1·40, 1·73) | <0·001 | 1·19 | (1·07, 1·33) | 0·001 |
| ≥40 kg/m^2^ | 1·58 | (1·40, 1·79) | <0·001 | 1·07 | (0·94, 1·21) | 0·318 |
