# Supplementary material for: Obesity and revision surgery, mortality, and patient-reported outcomes after primary knee replacement surgery in the National Joint Registry: A UK cohort study
Source: PLoS Med. 2021 Jul 16;18(7):e1003704. doi: 10.1371/journal.pmed.1003704 (PMC8284626; doi:10.1371/journal.pmed.1003704)
Supplement: S2 Table — Adjusted models adjusted for age, gender, ASA grade, year of primary TKR, indication for operation, IMD, and Charlson comorbidity index. ASA, American Society of Anaesthesiologists; BMI, body mass index; HES, Hospital Episodes Statistics; IMD, Index of Multiple Deprivation; NJR, National Joint Registry; TKR, total knee replacement. (DOCX) [file pmed.1003704.s006.docx]

|  | **Mortality model 1: univariable model** | | | **Mortality model 2: adjusted for age, sex, year primary TKR, ASA grade** | | |
| --- | --- | --- | --- | --- | --- | --- |
| **BMI** | **HR** | **95% CI** | **p-value** | **HR** | **95% CI** | **p-value** |
| <18·5 kg/m^2^ | 1·42 | (0·63, 3·21) | 0·402 | 1·44 | (0·64, 3·28) | 0·374 |
| 18·5–24·99 kg/m^2^ (reference) | 1·00 |  |  | 1·00 |  |  |
| 25–29·99 kg/m^2^ | 0·65 | (0·53, 0·79) | <0·001 | 0·75 | (0·61, 0·91) | 0·004 |
| 30–34·99 kg/m^2^ | 0·45 | (0·36, 0·55) | <0·001 | 0·63 | (0·51, 0·79) | <0·001 |
| 35–39·99 kg/m^2^ | 0·46 | (0·36, 0·59) | <0·001 | 0·78 | (0·61, 1·02) | 0·065 |
| ≥40 kg/m^2^ | 0·51 | (0·37, 0·70) | <0·001 | 1·00 | (0·71, 1·40) | 0·983 |
