# Supplementary material for: Obesity and revision surgery, mortality, and patient-reported outcomes after primary knee replacement surgery in the National Joint Registry: A UK cohort study
Source: PLoS Med. 2021 Jul 16;18(7):e1003704. doi: 10.1371/journal.pmed.1003704 (PMC8284626; doi:10.1371/journal.pmed.1003704)
Supplement: S3 Table — Adjusted model adjusts for age, gender, ASA grade, indication for operation, fixation type, and year of receiving the primary TKR, anxiety status, Charlson score, and multiple deprivation index. ASA, American Society of Anaesthesiologists; BMI, body mass index; OKS, Oxford Knee Score; TKR, total knee replacement. (DOCX) [file pmed.1003704.s007.docx]

|  | **Unadjusted** | | | **Adjusted model** | | |
| --- | --- | --- | --- | --- | --- | --- |
| **BMI** | **Coefficient** | **95% CI** | **p-value** | **Coefficient** | **95% CI** | **p-value** |
| <18·5 kg/m^2^ | -1·07 | (-2·11, -0·29) | 0·038 | -0·65 | (-1·66, 0·35) | 0·204 |
| 18·5–24·99 kg/m^2^ (reference) | 0·00 |  |  | 0·00 |  |  |
| 25–29·99 kg/m^2^ | -0·25 | (-0·42, -0·07) | 0·004 | -0·33 | (-0·50, -0·16) | <0·001 |
| 30–34·99 kg/m^2^ | -1·07 | (-1·24, -0·89) | <0.001 | -1·01 | (-1·19, -0·84) | <0.001 |
| 35–39·99 kg/m^2^ | -1·96 | (-2·16, -1·77) | <0.001 | -1·65 | (-1·86, -1·45) | <0.001 |
| ≥40 kg/m^2^ | -2·83 | (-3·08, -2·58) | <0.001 | -2·01 | (-2·27, -1·75) | <0.001 |
