# Supplementary material for: Obesity and revision surgery, mortality, and patient-reported outcomes after primary knee replacement surgery in the National Joint Registry: A UK cohort study
Source: PLoS Med. 2021 Jul 16;18(7):e1003704. doi: 10.1371/journal.pmed.1003704 (PMC8284626; doi:10.1371/journal.pmed.1003704)
Supplement: Attachment — Submitted filename: V1.2_response_to_reviewers.docx [file pmed.1003704.s008.docx]

18^th^ December 2020

Dear Ms Veitch,

**PMEDICINE-D-20-04558R1 - The effect of obesity on revision surgery, mortality and patient reported outcomes of primary knee replacement surgery: data from the National Joint Registry**

We thank you and the reviewers for your helpful reviews and comments on our manuscript. We have addressed your comments and queries as detailed below and have altered the manuscript accordingly. We would be very grateful for your further review for suitability for publication in PLOS Medicine.

Thank you very much again for your time,

Yours sincerely,

Jonathan T Evans on behalf of the authors

Academic Clinical Lecturer in Trauma & Orthopaedics

University of Bristol, UK

Requests from the editors:

**Please structure your abstract using the PLOS Medicine headings (Background, Methods and Findings, Conclusions); we also recommend authors include a brief note about any key limitation(s) of the study's methodology in the abstract, ideally in Methods and Findings subsection.**

Thank you, we have made these changes to the abstract. We have added the following statement regarding limitations to the Methods and findings section:

“The main limitations were missing BMI data particularly in the early years of data collection and a potential selection bias effect of surgeons selecting the fitter patients with raised BMI for surgery.”

**At this stage, we ask that you include a short, non-technical Author Summary of your research to make findings accessible to a wide audience that includes both scientists and non-scientists. The Author Summary should immediately follow the Abstract in your revised manuscript. This text is subject to editorial change and should be distinct from the scientific abstract. Please see our author guidelines for more information: https://journals.plos.org/plosmedicine/s/revising-your-manuscript#loc-author-summary**

**Author Summary**

**Why Was This Study Done?**

•	Whilst total knee replacements are generally considered safe and effective, it has been suggested that patients with high BMI are at increased risk of poor outcomes, leading to policies restricting who is referred for surgery.

•	Previous studies of the impact of BMI have used smaller datasets or have focussed on a single outcome rather than the wider focus of this article which includes mortality, implant survival and patient reported outcomes.

- We aimed to investigate whether patients with a raised BMI operated on within the National Joint Registry had demonstrably worse outcomes following total knee replacement.

**What Did the Researchers Do and Find?**

- We analysed 493,710 total knee replacements implanted between 2005 and 2016 to investigate the proportion of patients that died within 90 days, how many implants needed revising (re-do surgery) after 10 years and the changes between pre-operative and six-month post-operative Oxford Knee Score.
- Patients with raised BMI (according to WHO categories) were compared to those with a “normal” BMI.
- Patients in the “Overweight” and “Obese” groups had a lower 90-day mortality than those with “Normal” BMI.
- Total knee replacement in patients with raised BMI were more likely to have been revised after 10 years although the cumulative revision estimate in all groups was below the benchmark of 5% generally considered to be acceptable.
- All patient groups demonstrated an improvement in Oxford Knee Score after six months. The “Overweight” and “Obese” groups demonstrated a smaller relative improvement compared to the “Normal” group; however, this relative difference was below the threshold considered to be clinically meaningful.

**What Do These Findings Mean?**

- There does not appear to be any evidence to support clinically relevant worse outcomes following TKR for patients with a raised BMI in the National Joint Registry between 2005 and 2016.
- These findings do not support restriction of referral for knee replacement based on BMI alone. It appears that even if some patients with raised BMI are at risk of poorer outcomes, the outcomes remain acceptable by contemporary standards, the selection process of orthopaedic surgeons is effective at identifying the correct patients to operate on at a population level.

**If possible, please reformat the citation style into PLOS Medicine's format (should be straight forward if using referencing software) - this should use callouts formatted as sequential numerals in square brackets (not superscript).**

Thank you for highlighting this error, we have made this change.

**The authors might wish to look at established guidance for reporting studies conducted using routinely-collected datasets (which include some items related to record linkage between databases) - eg the RECORD guidance - https://www.equator-network.org/reporting-guidelines/record/; https://journals.plos.org/plosmedicine/article?id=10.1371/journal.pmed.1001885. If using that statement to report the study we'd suggest the authors note this in the Methods, citing the RECORD paper, and then also append as a supplementary file the completed RECORD checklist.**

Thank you for this helpful suggestion. We have now completed this and it is available in the supplementary information with the following text in the Methods section:

“Reporting of the study was in keeping with guidance provided in the Reporting of studies Conducted using Observational Routinely-collected Data (RECORD) statement (S1 RECORD Checklist). [23]”

**Can the authors clarify in the paper (methods section) whether the analysis reported here corresponded to one laid out in a prospective protocol or analysis plan? Please state this (either way) early in the Methods section.**

**a) If a prospective analysis plan (from your funding proposal, IRB or other ethics committee submission, study protocol, or other planning document written before analyzing the data) was used in designing the study, please include the relevant prospectively written document with your revised manuscript as a Supporting Information file to be published alongside your study, and cite it in the Methods section. A legend for this file should be included at the end of your manuscript.**

**b) If no such document exists, please make sure that the Methods section transparently describes when analyses were planned, and when/why any data-driven changes to analyses took place.**

**c) In either case, changes in the analysis-- including those made in response to peer review comments-- should be identified as such in the Methods section of the paper, with rationale.**

Thank you, the following statement has been added to the end of the Methods section.

“The analysis plan was made prior to the start of all analyses and agreed amongst co-authors. No data-driven changes to the analysis plan were made. An additional sensitivity analysis with BMI as a continuous variable using splines (at WHO cut-offs) to investigate nonlinearity was included in response to peer review.”

**In Discussion, "unanswered questions...", the following sentence seems to be lacking an additional phrase, see **xx** below.**

**"If it is accepted that BMI is not an appropriate rationing tool for TKR, then work looking at whether other instruments such as pre-operative OKS **assessments could be used**, may be useful.**

Thank you for highlighting this omission. We have included the following text:

“If it is accepted that BMI is not an appropriate rationing tool for TKR then work looking at whether other instruments such as pre-operative OKS assessments could be used, may be useful.”

**-----------------------------------------------------------**

**Comments from the reviewers:**

**Reviewer #1:**

**I confine my remarks to statistical aspects of this paper.**

**The general approach is fine and I like the use of new methods and the fact that the authors included all the covariates rather than doing some sort of model building.**

**However, I have a few issues to resolve before I can recommend publication.**

**The main issue is that BMI should not be categorized for the analysis (although the categories may be useful for some parts of presentation). Leave BMI as a number and investigate nonlinearity with a spline. The use of a spline on a categorized version of a variable is very limited. It limits estimation of knots - they have to happen at the cut-offs between categories. See *Regression Modelling Strategies* by Frank Harrell for more problems with categorizing continuous variables.**

Thank you very much for this suggestion. Given the size of the data set we felt that any loss of power from categorising BMI would be negligible. To support this decision and hopefully reassure you, we have now run the analyses with BMI as a continuous variable using splines in each of the analyses (mortality, revision and PROMs) as a sensitivity analysis. We now present these analyses in the article as well as in this response document for your information.

For the mortality and PROMs analyses, a likelihood ratio test comparing a model with BMI as a continuous variable to our existing model showed strong evidence against the null hypothesis of linearity, supporting our use of categorisation. We agree that the use of splines to split the continuous BMI according to WHO categories is an option and now present these analyses (see below). Given the sample size in this study, the similarity in results and the more complex interpretation of the model including splines our preference is to remain with BMI as a categorical variable in the results tables, but to now include graphs of restricted cubic splines to describe the linearity of effect. Presentation of the results using BMI as a spline variable is far more challenging and we feel makes it harder for a generalist audience to interpret, therefore our preference is for presenting a combination of both analyses.

The survival analyses (using flexible parametric models) suggested weak evidence against the null hypothesis of linearity. To maintain continuity with the other analyses we elected to continue with categorisation of BMI.

**Revision**

**Hazard of revision within 11 years of total knee replacement relative to patients with a BMI of 22.5 modelled using flexible parametric survival analysis using BMI as a continuous variable with restricted cubic splines at cut offs of WHO criteria. Model adjusting for age, sex, ASA grade, indication for operation, year of primary TKR and fixation type.**

|  | **Coefficient** | **95% CI** | **P** |
| --- | --- | --- | --- |
| Spline 1 | 1.05 | 1.03, 1.07 | <0.0001 |
| Spline 2 | 1.01 | 0.99, 1.03 | 0.265 |
| Spline 3 | 1.00 | 0.98, 1.02 | 0.907 |
| Spline 4 | 1.01 | 0.99, 1.03 | 0.454 |
| Spline 5 | 1.01 | 0.99, 1.03 | 0.529 |
| Spline 6 | 0.98 | 0.96, 1.00 | 0.018 |

**Mortality**

**Hazard of death within 90 days of total knee replacement relative to patients with a BMI of 22.5 modelled using Cox proportional hazards using BMI as a continuous variable with restricted cubic splines at cut offs of WHO criteria. Model adjusting for age, sex, ASA grade, indication for operation and year of primary TKR.**

|  | **Coefficient** | **95% CI** | **P** |
| --- | --- | --- | --- |
| Spline 1 | 1.05 | 0.99, 1.12 | 0.074 |
| Spline 2 | 0.85 | 0.81, 0.89 | <0.0001 |
| Spline 3 | 0.99 | 0.85, 1.02 | 0.566 |
| Spline 4 | 1.04 | 0.99, 1.09 | 0.108 |
| Spline 5 | 1.05 | 1.00, 1.11 | 0.062 |
| Spline 6 | 1.02 | 0.96, 1.07 | 0.543 |

**PROMs**

**Change in mean Oxford Knee Score six months after total knee replacement relative to patients with a BMI of 22.5 modelled using linear regression using BMI as a continuous variable with restricted cubic splines at cut offs of WHO criteria. Model adjusting for age, sex, ASA grade, indication for operation, fixation type, year of primary TKR and anxiety status.**

|  | **Coefficient** | **95% CI** | **P** |
| --- | --- | --- | --- |
| Spline 1 | -0.69 | -0.75, -0.63 | <0.0001 |
| Spline 2 | -0.01 | -0.64, 0.04 | 0.608 |
| Spline 3 | -0.12 | -0.17, -0.08 | <0.0001 |
| Spline 4 | -0.13 | -0.18, -0.09 | <0.0001 |
| Spline 5 | 0.01 | -0.04, 0.06 | 0.658 |
| Spline 6 | 0.04 | -0.01, 0.09 | 0.144 |

**Grouping age has some of the same issue, but the authors use so many groups that it's probably OK. Still, why not leave age as "years"?**

Yes, we agree with this concept in a similar way to above. However, in this case as BMI is the exposure of interest and age is a confounder, given the large sample size we feel that using age as a categorical variable with many groups does not cause a loss of precision.

**Why was year grouped the way it was? That would seem to force year to be categorical and would waste degrees of freedom (not really a big problem here) and make interpretation of any year effect harder.**

The decision to group year is based upon the knowledge that survival of TKRs varies by date of implant with more recently performed operations appearing to have greater survival. The NJR started collecting data on TKR in 2004 and BMI in 2005 and as such it took a couple of years to build high numbers and completeness of data. Data on TKRs entered prior to the date that the NJR commenced collecting BMI data were excluded. Therefore, splitting 2005-2007 into individual years may cause the problems discussed above regarding sub-group analysis and loss of power. We felt that by 2008 numbers were sufficient to split by individual year.

**Fig 2 The y axis should be rescaled to something like 0 to 5 or 6. As is, most of the graph is blank. This makes it harder to see the rela** *[n.b. reviewer comment cuts off]*

We agree, and the figure has been reformatted. See below:

**Fig. 3. I don't like that the scales of the two axes are so different. The plot makes it look like there was equal improvement for all initial conditions, when, really, there was no improvement at the highest initial levels. I suggest either making both axes go from 0 to 50 or else a graph with initial score on the x axis and improvement on the y axis. This seems very important. The authors note that BMI isn't a useful predictor of success (and, as an overweight man who will probably need knee surgery, I appreciate that!) but another useful finding here (which might make another paper) is that people with good initial OKS didn't really improve much.**

Thank you. We have made both changes as suggested and are happy to put either graph in the manuscript (for now we have used the post-op score on the y axis). Since submission of this article, further research has come from another group regarding OKS and selection for TKR, so we have not made any edits based on this suggestion [1].

1. Price AJ, Kang S, Cook JA, Dakin H, Blom A, Arden N, et al. The use of patient-reported outcome measures to guide referral for hip and knee arthroplasty. The Bone & Joint Journal. 2020;102-B(7):941-9.

**Peter Flom**

**-----------------------------------------------------------**

**Reviewer #2:**

**The authors have written a clear and logically constructed article suggesting the higher body mass index is associated with slightly higher revision rate but similar clinical improvement (in terms of Oxford Knee Score) after six months after primary knee replacement. The case numbers are clearly sufficient and the follow-up long enough to report revision rates. Nationally representative materials and appropriate statistical methodology can be also considered as strengths of this study. Unfortunately, there are also several issues to criticize. However, I believe the authors have good possibilities to satisfactorily respond to these concerns.**

**Major concerns:**

**1) I have troubles following the description of collection of the data and creating the datasets. I understand that revision data and clinical data come from different sources and are from different time periods but presenting four (!) different flowcharts is confusing. Figure 1 of the manuscript is quite ok although I do not understand why to report the total number of TKRs since 2003 although included cases were from 2005-2016. I am also able to follow Figure 1 in the supplementary materials but do not understand the next flowchart Figure 2 at all (it ends up with figure 288,423 that is not mentioned anywhere else) and the last flowchart feels like repetition. Also, the different time periods are confusing and poorly explained acronyms (LM, HES) complicate interpretation. The authors should bear in mind that most readers are likely not familiar with the registers of the authors' country.**

Thank you for raising this in order to provide clarity, we have started from scratch again with the flow charts. The previous versions were detailed and presented too much information in a way that was hoped would be comprehensive, but we appreciate it has instead led to confusion. We hope that Fig 1, where we have combined the necessary information, is clearer and more informative.

**It is noteworthy that there are lots of missing data. About one third of cases were excluded because of missing BMI and there were exclusions for other reasons as well. I do not quite agree with the authors that excluded patients were comparable to the included ones. According to table 1, there were more patients with lower ASA and more cementless TKRs in the incomplete (i.e. excluded) group.**

We have added the following text to the manuscript:

“The main differences between groups (Table 1) were the distribution of patients between the ASA 1 and 2 groups and fixation type. Results of patients with ASA 1 and 2 tend to be similar and so we do not feel this is likely to have biased results. More patients missing BMI had cementless or hybrid fixation compared to those with BMI reported. Given the NJR annual report suggests poorer implant survival in cementless TKR [3], this difference could result in reduced survival overall and depending on how BMI is distributed amongst high BMI patients could bias our results either way although these fixation methods are only used in a small proportion of patients (4.1% of those with complete data and 7.6% of those with incomplete data).”

**Differences e.g. in comorbidity, type of operating unit and social status are not reported. It is also likely that patients responding to Oxford Knee Score represent a selected sample of patients (younger, uncomplicated and so on), limiting generalizability of the findings).**

Thank you for highlighting this. These details were not included in Table 2 as data on co-morbidity and social status (IMD) are part of a sensitivity analysis and therefore using a smaller data set (see new Fig 1) and their addition into Table 2 would make the table more confusing. Considering these variables are not part of the model for the primary outcomes we have elected not to produce a further table on the smaller linked data set.

Regarding non-responders to PROMS questionnaires, a previous study using a similar data set reports that male, younger patients who did not live alone were less likely to respond. This article also suggested that those with longer mean duration of symptoms and with one or more co-morbidity were less likely to respond. Whilst these differences reached “statistical significance” they represented small absolute differences and, we feel, are likely to have little impact on overall results affecting generalisability of findings [1]. These findings are also reflected in an earlier study [2].

1. Edwards HB, Smith M, Herrett E, MacGregor A, Blom A, Ben-Shlomo Y. The Effect of Age, Sex, Area Deprivation, and Living Arrangements on Total Knee Replacement Outcomes: A Study Involving the United Kingdom National Joint Registry Dataset. JB JS Open Access. 2018;3(2):e0042.
2. Hutchings A, Neuburger J, Grosse Frie K, Black N, van der Meulen J. Factors associated with non-response in routine use of patient reported outcome measures after elective surgery in England. Health Qual Life Outcomes. 2012;10:34.

**These issues related to possible selection bias should be reported and discussed more thoroughly. Especially the results about lower mortality with higher BMIs indicate that in the obese groups there have been significant patient selection (and/or differences in pre- and perioperative care) that are not fully captured by the variables used in this study.**

Thank you for highlighting this. We have discussed this issue in further detail and focussed on the fact that current processes in place for patient selection seem to be effective therefore meaning that additional restriction of referral based on BMI is unlikely to be of use:

“The data available in our study did not allow this to be explored in more depth. Individual units or surgeons may employ different methods of determining a patient’s fitness for surgery as well as differing pre- and post-operative care for these patients. Our results do suggest that the processes already in place are suitable identifying those high BMI patients at increased risk of death and that restricting access to surgery at the point of referral is unlikely to be of benefit.”

**2) Validity and coverage of the data sources should be described. Although national registers usually cover primary joint replacements quite well, the coverage of revision joint replacements is usually poorer. From the viewpoint of the current study, prosthetic-joint infections represent a specific problem as their risk is higher in patients with highest BMI, and they can be managed with other means than full revision surgery especially in high-risk patients and in acute infections. One might have expected higher early revision rate in the most obese group because of PJIs.**

Thank you for this comment. The 14th NJR annual report (2017) describes the results of the first NJR data quality audit, suggesting that approximately 95.7% of primary TKRs and 90.3% of revision TKRs were recorded in the NJR for financial year 2014/15. The NJR records revision as any procedure where an implant is added, removed, or modified. Patients who underwent a Debridement and Implant Retention (DAIR) operation where there was not exchange of any modular components for suspected/confirmed infection prior to 2019 would not be captured by the NJR (since 2019, DAIR without modular exchange is now captured). Those where a DAIR was performed with exchange of modular components were captured as single stage revision procedures. Non-revision procedures are not captured by the NJR This is likely to represent a small number however we have included a statement regarding this in the discussion:

“As with all registry data, analyses are only as good as data entered, the first NJR data quality audit suggested that 95.7% of primary TKRs and 90.3% of revision TKRs were captured in financial year 204/15. Despite this high level of completeness, at the time of data collection the NJR did not routinely capture operations where implants were not added, removed, or modified. This means that if a patient returned to theatre for an operation that did not involve the change of any implants, it wouldn’t have been captured by the NJR and would therefore not be reported by our study. ”

**It is unclear how data about different confounding variables were collected, what is their completeness and coverage. Most readers may not be familiar with Multiple Deprivation Index and EQ5D, the coverage of latter of which could be expected to be limited.**

Age, Sex, BMI, Indication, ASA, date of operation and mode of fixation were all provided directly from the NJR. The Index of Multiple Deprivation (IMD) was provided by the HES data set. The EQ5D was collected directly from National PROMS data that was subsequently linked to HES records. At your suggestion we have included a brief explanation of the IMD in the manuscript:

“The Index of Multiple Deprivation is the official measure of relative deprivation for small areas (Lower Layer Super Output Areas) in England. The measure is calculated using seven domains including income, employment, education, health, crime, and environment. It ranks every small area from 1 (most deprived to 32,844 (least deprived).”

**3) The materials were collected until December 31, 2016, and the same date was the end of follow-up. Consequently, some of the patients had practically zero follow-up and they could not have experienced the outcomes that were of interest. Although for example in the analysis of deaths, Cox analysis (taking into account different follow-up times) was used appropriately, including also patients without sufficient follow-up in the denominator leads to falsely low absolute rates of e.g. deaths or revisions. Now that it is already year 2020, it is difficult to see, why the authors have not used more later data - or in the other hand, made the choice of materials so that all patients would have reasonable minimum follow-up (at least six months to get PROM data or more preferably one year).**

This is a recognised issue with longitudinal data and is the rationale behind the use of survival analysis techniques rather than proportions in the reporting of implant survival or mortality. As you state, Cox proportional hazards modelling does take this into account in our mortality analyses and the same is true for both crude and adjusted cumulative revision estimates. The crude analyses (seen in Table 3 and S4 Fig) use Kaplan-Meier and adjusted analyses (seen in Fig 2 and Tables 4 and 5) use Flexible Parametric Survival Analysis, both of which account for right censoring of data. Regarding denominator numbers, S4 Fig includes an “at risk” table demonstrating the number of TKR still in situ at time of reporting. Lettin et al. suggest in their 1991 article that the number of TKRs that must remain in place to allow reliable estimates from survival analysis is 40 [1]. After 10 years, the only sub-group in our analyses that does not have 40 patients at risk is the underweight patients with all other groups having over 900 TKRs at risk. We do agree that in an ideal world including the most up to date data would allow longer term reporting with more patients at risk, however on this occasion we are limited by the fact our data access agreement for this research topic specified data from the NJR up to 31^st^ December 2016 and as such we cannot extend this.

1. Lettin AW, Ware HS, Morris RW. Survivorship analysis and confidence intervals. An assessment with reference to the Stanmore total knee replacement. The Journal of bone and joint surgery British volume. 1991;73(5):729-31.

**There are also discrepancies in the observation period: methods section say that cases were included from 2005-2016, but flowcharts in the supplementary materials and results section also talk about period 2009-2014.**

Thank you for highlighting this, we apologise for the lack of clarity; the difference relates to which data were available for analysis. Survival and mortality outcomes were available directly from the NJR database and as such were available to us until 31^st^ December 2016. The PROMs data were accessed via the Health Episodes Statistics (HES) database. These data were only available to us until 30^th^ December 2014. We recognise that we had not made this clear enough in the Methods section and have now amended this:

“Linkage between PROMs and the NJR was made via the Health Episodes Statistics database, which records details of all hospital admissions in England (S1 Fig) using the same exclusion criteria. HES data and subsequently PROM data were only available up to the 30th December 2014.”

**The adjustments used in the regression models are also described incompletely and/or contradictory: methods section lists several confounding factors but not all of them were used in all analyses, according to legends of 4-6 and the tables presented in supplemental materials. Most materials of supplementary materials are not referred to in the text. As there have been several multivariable models, statements like "fully adjusted model" are not accurate enough.**

We agree that the term fully adjusted model is confusing in the tables. We have now changed this to “Adjusted model” and ensured that the caption for the table details exactly which co-variates were included in the model.

**4) I'd appreciate more critical analysis and interpretation of the results and more thorough review of the literature.**

**- With all due respect, now the manuscript has a feeling that its aim is to support the view that BMI should not have a role in rationing knee replacement although earlier literature and also the current results might be interpreted to support the opposite view. There are a couple of recent meta-analyses (indicating poorer results with higher BMI) that have been ignored (Chaudhry et al. JBJS Rev 2019, Sun & Li Knee 2017, Si et al. Knee Surg Sports Traumatol Arthrosc 2015) - and considering numerous earlier reports, it should be articulated more clearly what are the news that the present study give (mid-term revision rates could be such). The present study is somewhat contradictory with earlier meta-analyses and reasons for these discrepancies should be commented.**

Thank you for highlighting these interesting articles to us. We are aware of the conflicting messages and did briefly discuss this in our discussion. In view of your suggestion, we have gone slightly further and drawn further direct comparison to Chaudry et al.:

“Our findings are generally conflicting with those of Chaudry and colleagues, who in 2019 published a meta-analysis suggesting higher risk of revision and worse patient reported outcomes in “severely, morbidly and super-obese patients” [31]. The main limitation of their analyses was the quality of included studies. Their conclusions focussed on revision rate being driven by septic revisions, a sub-group we did not specifically look at in our study. Similarly, to Chaudry et al, we reported an increased revision risk in patients with higher BMI but concluded the cumulative revision estimate was still below the nationally recognised benchmark.”

**- Early complications (not leading to revision joint replacement), possibly prolonged hospitalizations and particularly prosthetic-joint infections have not been taken into account in the present analysis, which should be discussed as a limitation as they affect the costs and hence cost-effectiveness and rationing of surgery.**

Thank you, this issue was also raised by reviewer 1 and we have added text into the manuscript to address this. We did not investigate length of stay as an outcome in this study as you state, and we have added in a comment to the limitations as you suggest.

“As with all registry data, analyses are only as good as data entered, the first NJR data quality audit suggested that 95.7% of primary TKRs and 90.3% of revision TKRs were captured in financial year 204/15. Despite this high level of completeness, at the time of data collection the NJR did not routinely capture operations where implants were not added, removed, or modified. This means that if a patient returned to theatre for an operation that did not involve the change of any implants, it wouldn’t have been captured by the NJR and would therefore not be reported by our study.”

“We did not investigate factors such as length of stay, which may have an impact on cost-effectiveness or primary TKR in this study. If BMI changes length of stay it may lead to increased costs therefore future studies could investigate the effect of cost-effectiveness as an outcome.”

**Also, one further reason supporting conservative treatment rather than surgery is that weight loss and rehabilitation have been reported to ease the symptoms of osteoarthritis, making knee replacement possibly unnecessary at least for a while.**

Whilst this is undoubtedly true for some patients who are amenable to such a management strategy, it is not the focus of this article and further investigation of this is beyond the scope of this study. In view of word count, we have elected not to further comment on this in the manuscript, however, are happy to discuss this point at the editor’s discretion.

**- The authors suggest that reporting revision rates, mortality and clinical outcome all in the same study is a strength of this study. Combining the three but analyzing them separately however does not necessary give any extra over separate and possibly more detailed studies. In the Discussion, the authors write "The failings of examining single domains have previously been highlighted, in that just because a TKR has not been revised does not necessarily mean it was a success." to reason combining the three outcomes. Because the present study analyzes clinical outcome after six months but revision rates up to 10 years, it does not overcome this problem, and problem with falsely low revision rate as a result of not performing revision in hight risk patients still remains a problem and should be acknowledged.**

We understand the reviewer’s viewpoint regarding this point. We feel that combining the three domains is a strength in terms of efficiency and generalisability but agree that a reader may wish to consider each research question independently. This study uses the largest national arthroplasty registry in the world and includes more patients at longer follow up times than previous studies in the same area, even if conclusions are similar, we feel this study adds to the body of evidence both in triangulation of signals as well as generalisability.

With regard to the final comment, we agree and have included the following statement into the limitations.

“It is possible that patients may require revision surgery but are deemed unsuitable because of co-morbidities and as such are not identified by the NJR as a failure. Whilst this is a recognised limitation of registry research, it may be particularly relevant in this study, if patients with high BMI at the time of primary surgery are considered higher risk of developing future co-morbidities that would render them less fit for revision surgery.”

**Minor concerns:**

**- References 8 and 9 are about hip replacements although there are suitable ones about knees as well.**

Thank you for highlighting this, we have now amended the referencing to refer to revision total knee replacement articles.

1. Kallala RF, Vanhegan IS, Ibrahim MS, Sarmah S, Haddad FS. Financial analysis of revision knee surgery based on NHS tariffs and hospital costs: does it pay to provide a revision service? Bone Joint J. 2015;97-B(2):197-201.
2. Weber M, Renkawitz T, Voellner F, Craiovan B, Greimel F, Worlicek M, et al. Revision Surgery in Total Joint Replacement Is Cost-Intensive. Biomed Res Int. 2018;2018:8987104.

**- Age is a continuous variables. Why is it used as classified one?**

We recognise this potential limitation of categorising age and it was also raised by reviewer 1. As discussed above, due to the very large numbers included in this study (a feature unique to large registry analyses such as this) categorisation results in very large groups and as such there is no noticeable loss of power. This issue, particularly regarding BMI is discussed in detail in response to the comments of reviewer 1.

**- How (staged or same-day) bilateral knee replacements were dealt with? This is relevant both concerning the outcome analysis but also data linkage.**

Bilateral knee replacements were handled differently according to the different research questions asked. We have added a statement into the manuscript to make this clear:

“For survival outcomes each knee replacement was treated as an individual, this is possible given the nature of reporting of both primary and revisions in the NJR. For PROMS and mortality analyses however, same day knee replacements could not be interpreted individually. For this reason, in same day TKRs only one was selected at random to contribute to the analyses to avoid duplication of data.”

**- Fulfilment of proportional hazards assumption should be commented in association with use of Cox regression analysis.**

Thank you for highlighting this omission from the text. We used several techniques to check proportionality of hazards for the Cox regression used for mortality analyses. We assessed Schoenfeld residuals as well as plotting a log(-log(survival)) versus log of survival time graph (see below). We have now included detail of this in the manuscript.

“The assumption of proportionality of hazards was assessed visually and through the use of Schoenfeld residuals.”

**-**

**Please, keep the order of analyzed outcomes the same throughout the manuscript (now OKS is mentioned first in the results section).**

Thank you for highlighting this, we have now corrected this, and restricted mention of the OKS results to the correct section.

**- What does BMI <0 mean?**

I believe this comment is referring to the original Fig 1. This refers to TKRs excluded from analyses due to inconsistent or unrealistic data. BMI that has been entered as under 0 (i.e. a negative value) or over 60 is incoherent and therefore added in error and therefore treated the case as if it were missing. This has been clarified in the new Fig 1.

**- Y-axis in Figure 3 should start from zero or it should be indicated that it is cut.**

Thank you, in keeping with comments from reviewer 1 this figure has been edited. We hope the new version is more acceptable.

**- It seems that Figure 3 represents data from a linear model. In its current form its value is questionable and interpretation is difficult: I think it is not that certain that association is linear but instead from lower scores the improvement might be greater. Ceiling effect may also be present. Secondly, there is possibly variation in individual patients' responses and one might hypothesize that variation might be greater in the obese groups. I'd suggest replacing Figure 3 with information based on actual observations (change from pre to postop in different BMI categories and its variation).**

You are correct, the PROMs model is a linear model, as stated in the methods section. In keeping with another reviewer’s comments, we have amended Fig 3 (see below). We agree that the cropping of the Y axis inadvertently made the relationship appear different to the one observed. As you can see from the new figure, the improvement in OKS was smaller as pre-operative score increased.

**- I's suggest using Kaplan-Meier analysis (instead of modelled curves of Figure 2) in the manuscript as results from adjusted models are already shown in the tables.**

At the moment we present the Kaplan-Meier results in the Supplementary information (S4 Fig) but would fully support including this in the main manuscript at the editor’s discretion if space is available.

**- Although the change in OKS in different BMI groups is within minimal clinically meaningful difference, it is noteworthy that the differences in absolute OKSs are greater than 4 and remain essentially unchanged after operation.**

We agree, Table 3 demonstrates that the improvement is similar across all groups, but the final OKS achieved is lower in the higher BMI groups (where the starting BMI was lower). The primary outcome for this study was the change in OKS as a marker of whether the operation was effective. We have however included a comment in the manuscript to note your point:

“The six-month absolute OKS appeared lower in higher BMI categories relatively, which reflects a lower starting point in these categories.”

**- 90-day mortality is reported as the outcome of interest, but also 30-day and 60-day mortality are reported in Table 3. Please, select which are the outcomes and at which time points they are analyzed. As it comes to mortality, I understand that 90-day mortality can be considered to represent the risk related to surgery. When weighing the value of surgery, also mortality in longer term is relevant: the benefit of surgery could be interpreted different e.g. if the most obese patients had high 5/10-year mortality. Furthermore, if there are significant differences in longer-term mortality, it may bias K-M and Cox analyses and one might argue that compering risk analyses should be used instead.**

Our primary outcome was 90 days for the reason suggested by the reviewer. The additional 30- and 60-day mortality results were included in Table 3 in a similar way to cumulative revision estimates at 3, 5 and 7 years. We agree that table 3 is very data heavy in its current format and as such we would be happy to remove all but the stated primary outcomes at the editor’s discretion. We disagree with the reviewer regarding the use of competing risks analysis in preference to Kaplan-Meier, we believe the two techniques are not biased in comparison to each other but instead answer different questions. This is a point we discuss in an article we published in 2018. (Sayers A, Evans JT, Whitehouse MR, Blom AW. Are competing risks models appropriate to describe implant failure? Acta Orthopaedica. 2018;89(3):256-8.)

**- I do not consider reporting p values necessary when HRs are shown with 95% CI.**

We are happy to present the HR either with or without P values at the editor’s discretion. We have checked previous studies of similar design and it would appear to be the journal’s preference to present both the 95% CI and p values.

**- Please replace p=0.0000 with p<0.001 as the p(probability) is hardly zero.**

Thank you for highlighting this. This was a result of transferring values from Stata and we had clearly missed some of the values. We have now corrected this to read <0.0001.

**- Supplemental figure 1: There are 252,659 PROMs-HES with unique NJR index numbers, of which ca. 15000 disappear after linkage (there are just 237,288 at the final step). This is not explained or illustrated satisfactorily (a box indicating the faith of these 15000 cases should be added).**

Given the comments of all reviewers we recognise the flow charts contained too much detail and were overly complex and hard to follow. We have now replaced these with a simpler version. The 15,371 cases you refer to, represent patients who had filled in PROMs and had an NJR number listed on HES but were not linkable to an NJR record.

**-----------------------------------------------------------**

**Reviewer #3:**

**This is a very interesting study of outcomes of TKA for obese patients. It is highly relevant for all the reasons highlighted by the authors. Also outside the UK, we see financially motivated attempts to reduce the number of operations, some of which appear entirely unjustified, e.g. requirements for months of physiotherapy in cases of OA with severe deformity, or refusing private cover for patients with a BMI over 35.**

**A register is not able to provide answers on causative relations, as the authors correctly argue, and the true effect of a factor on the outcome of a procedure is probably beyond the ability of registers. They are, however, one of the best means of getting information on current practice and current relations between possibly causative (and possibly confounded) factors and outcome. And no better starting point than the largest register.**

**The results are important, and the consequences of this study are potentially wide-reaching with general effects on health policy.**

**Does the NJR have information on postoperative weight (and weight loss)? If so, it would be very interesting to have the information included in the analyses.**

Thank you for your kind words. Unfortunately, the registry does not have information on post-operative weight or weight loss, so we are sadly unable to include this.

**Introduction**

***	Very good introduction. Although the aim appears well described, there is some uncertainty, that influences the judgement of appropriateness of the methods used. For instance: is there any time frame for the PRO outcome? Does the study aim to determine PRO improvements in the recovery period or the plateau outcome? Or is this a pragmatic analysis of the available data?**

Thank you, we agree the introduction was not very specific about the outcomes in particular the PRO. The PRO analysis was aimed at investigating change in Oxford Knee Score between pre-operative and six-month post-operative scores, this was a pragmatic decision based on the knowledge we had on what data would be available as collected by the National PROMs programme in the same setting as the NJR. We have amended the text to read the following to made time frames more obvious.

“Using data from the world’s largest joint replacement registry, our aim is to describe the association of BMI at the time of surgery with revision after 10 years, 90-day mortality and patient reported outcomes six months following primary TKR, and to consider the clinical importance of any observed association.”

**Methods**

***	Why were patients excluded, if the indication for revision was unknown? Revision is revision, and since indications may be less reliable, I would include all types of revision.**

This was a typographical error that had somehow passed through all our own checks. Thank you very much for highlighting it. Cases were excluded if indication for primary was unknown (162 cases). We hope this is clearer on our new flow chart.

***	Why was the analysis restricted to TKR? No interest in unicompartmentals?**

Whilst unicompartmental knee replacement (UKR) is certainly an interesting subject, this particular study is as you say, focussed on TKR. It is widely accepted that UKR may have different indications, be used in a population with different demographics and behaves in a different manner to TKR, in view of this, we felt that this would need a completely different study and was best not included within this article as a sub-group analysis.

***	The definition of revision is the well-known triad of removal, addition or exchange. Since a higher infection in obese patients has been suggested, and since some early infections may result in a reoperation not qualifying for a revision, some early complications may have been missed. This should be discussed. There may well be a general need to change focus from revisions to all types of reoperations, even if this deviates from tradition.**

Thank you very much. This point was similarly made by reviewer 2. We have included some new text to cover this in the limitations section.

“As with all registry data, analyses are only as good as data entered, the first NJR data quality audit suggested that 95.7% of primary TKRs and 90.3% of revision TKRs were captured in financial year 204/15. Despite this high level of completeness, at the time of data collection the NJR did not routinely capture operations where implants were not added, removed, or modified. This means that if a patient returned to theatre for an operation that did not involve the change of any implants, it wouldn’t have been captured by the NJR and would therefore not be reported by our study.”

***	It is not correct to suggest that the OKS covers 12 domains. The questionnaire was developed as a one-domain questionnaire (knee function and pain, not clearly separated), and it resulted in 12 questions within that domain.**

Thank you for the astute comment. We have now corrected this to the text below:

“The OKS is a patient-completed questionnaire which assesses knee pain and function with twelve questions, each rated scored from 0 to 4, completed using Likert scales, and the scores are summed to give a score from 0 (worst) to 48 (best) .[20].”

***	Had the authors had long-time PRO-data, one might argue for not excluding patients after revision. With a 6-month PRO time-frame, the exclusion appears justified.**

We agree with the reviewer that the long-term PROs comparing these groups would be interesting. This analysis was however, beyond the scope of this study but may prove to be a useful suggestion for a future project, thank you.

***	The reference to Royston and Parmar should be listed in the references. I am not an expert on the model used, and I would suggest a statistical review of the appropriateness of the model.**

Thank you for making this point. We have now included this reference within the statistical methods.

***	Is BMI data entered directly into the NJR, or is BMI calculated from entries of bodyweight and height? I would expect more errors with the former entry type. Please specify.**

On the NJR data entry form, the surgeon has the option of entering height and weight separately or as a BMI directly. If both are completed then the directly entered BMI is given precedence.

***	Is there no anchor question in the NJR? Would be very interesting to see this in relation to the OKS improvements instead of a simple reporting of the crude OKS improvements. See comment under Discussion below.**

The outcomes of the NJR do not include patient reported measures but are limited to binary measures of whether the knee has been revised or not and whether the patient is alive. The OKS data is collected by the National PROMs programme, which is administered by NHS Digital and the data subsequently linked to NJR data. As such there is unfortunately no anchor question to compare against the OKS improvements. This assumes that lack of revision is equal to success, which is a well-recognised limitation of the registry and something we discuss in the manuscript. It is also an important reason for us choosing to assess a range of outcomes in this study using OKS linked to those with NJR records.

**Results**

***	Comparing the complete and incomplete datasets, the ASA distributions do differ significantly, although possibly similar(?). The same is the case for fixation type. Possible reasons? Consequences? Comments?**

This question was also asked by an earlier reviewer and we have added some text into the manuscript to reflect this:

“The main differences between groups (Table 1) were the distribution of patients between the ASA 1 and 2 groups and fixation type. Results of patients with ASA 1 and 2 tend to be similar and so we do not feel this is likely to have biased results. More patients missing BMI had cementless or hybrid fixation compared to those with BMI reported. Given the NJR annual report suggests poorer implant survival in cementless TKR [3], this difference could result in reduced survival overall and depending on how BMI is distributed amongst high BMI patients could bias our results either way although these fixation methods are only used in a small proportion of patients (4.1% of those with complete data and 7.6% of those with incomplete data).”

***	Last 3 lines of Revision paragraph: It should be emphasized, that the 30-35 kg/m2 group did not differ significantly from the normal BMI group. To the casual reader, the message is an 8% higher revision risk, which may not be true.**

Thank you for highlighting this. We have made it clearer to the reader by specifically mentioning that the confidence interval for that category cross the null value of 1.

“The fully adjusted model shows that patients with BMI 30–34.99 kg/m2, 35–39.99 kg/m2 and ≥40 kg/m2 were 8% (HR 1.08 95% CI 0.99, 1.18), 21% (HR 1.21 95% CI 1.10, 1.32) and 13% (HR 1.13 95% CI 1.02, 1.26) more likely to undergo a revision than patients with BMI 18.5–24.99 kg/m2 respectively although it should be noted that the confidence intervals for the 30–34.99 kg/m2 category do cross the null value.”

***	First paragraph on OKS: This is just a repetition of table 6. Please provide an interpretation of the data.**

Thank you for pointing this out, we have adapted the text to interpret the coefficients rather than just repeat them:

“The increase in OKS (between pre-operative and six-month post-operative) for patients with raised BMI was smaller relative to patients with a “normal” BMI (table 6).”

**Discussion**

***	A possible limitation is the use of the OKS. The instrument was developed for knee OA patients, but as far as I remember, there was no mentioning of BMI for the focus group patients in the development paper. I also do not remember a validation paper for obese patients. The importance of this is that obese patients may have other needs and expectations, and the quantification of their feelings and function may well be biased by using the same instrument for both groups. As an example, I would assume that obese patients would have far more difficulty in kneeling (question 7) than non-obese patients, but this does not necessarily imply a problem with the surgical outcome. This should be discussed.**

The reviewer raises an important point here which is key to the interpretation of results, thank you. Original and subsequent validations of the OKS include a range of patients, some of which will have been of higher BMI but as far as we are aware, the score has not been validated specifically on patients with raised BMI. We have included some text in the manuscript to highlight this to the reader:

“Whilst the OKS has been widely validated it has not been specifically validated in a solely high BMI group. This could potentially create some bias in comparison of sub-groups of BMI if those with high BMI are more likely to score certain questions either higher or lower than patients with normal BMI.”

***	The timing of the post-operative OKS is a certain limitation that should be discussed. By comparing the two groups of patients at a single and identical time point assumes knowledge of identical improvement curves for the groups. It may well be that obese patients recover more slowly than non-obese patients, and the 6-month comparison may give a biased estimate of the "true" difference in outcome between the groups. One group may be measured in the recovery phase, while the other is measured in the plateau phase. This should also be discussed.**

This is a well-made point, and we agree. Post-operative OKS in this study was taken directly from the national PROMS programme and as such we were limited to six-month OKS. Given the change scores are similar between groups it would seem unlikely that further changes were likely to happen. We have however added a comment into the manuscript to highlight this potential source of bias:

“It is possible that recovery trajectories could vary according to BMI (i.e. higher BMI patients taking longer to recover). This could mean that patients in one particular group may not have achieved their peak post-operative outcome score by the six-month point reported in this study.”

**Figures**

**1.	It hardly seems necessary to specify reasons for exclusion of no patients.**

We agree with your comment and it was as a result of a standard set of exclusions we have used in previous studies. The flow charts have now been re-formatted in keeping with another reviewers comment and this problem resolved.

**2.	Y-axis should be rescaled to better visualize differences between curves, e.g. range 0-5%.**

Thank you for this comment which has been made by other reviewers. The figure has been amended as below:

**3.	A graphical representation of the uncertainty of the estimate curves would increase the value of this figure. It would be difficult to represent all five, but a cumulated model could be shown.**

We agree this would be useful, however as the reviewer points out it would be difficult to represent all five. At the editors request we are happy to produce a cumulative estimate with confidence intervals instead of the new figure 3 produced at the request of reviewer 1.

**Nov 10, 2020**

**Anders Odgaard**

**Rigshospitalet, Copenhagen University Hospital**
