# Supplementary material for: Obesity and revision surgery, mortality, and patient-reported outcomes after primary knee replacement surgery in the National Joint Registry: A UK cohort study
Source: PLoS Med. 2021 Jul 16;18(7):e1003704. doi: 10.1371/journal.pmed.1003704 (PMC8284626; doi:10.1371/journal.pmed.1003704)
Supplement: Attachment — Submitted filename: V1.0_response_to_reviewers_2.docx [file pmed.1003704.s009.docx]

15 June 2021

Dear Mr Turner,

**PMEDICINE-D-20-04558R2 - The effect of obesity on revision surgery, mortality and patient reported outcomes of primary knee replacement surgery: data from the National Joint Registry**

We thank you and the reviewers for your helpful reviews and comments on our revised manuscript. We have addressed your comments and queries as detailed below and have altered the manuscript accordingly. We would be very grateful for your further review for suitability for publication in PLOS Medicine.

Thank you very much again for your time,

Yours sincerely,

Jonathan T Evans on behalf of the authors

Academic Clinical Lecturer in Trauma & Orthopaedics

University of Bristol, UK

**Requests from the editors:**

**Bearing in mind the observational research design, we ask you to amend the title to better accord with journal style, and suggest: "Obesity and revision surgery, mortality and patient reported outcomes after primary knee replacement surgery in the National Joint Registry for England, Wales, Northern Ireland and the Isle of Man: A cohort study".**

Thank you for the suggestion. We have made the change as suggested. During the review process the National Joint Registry has changed its name to make it less cumbersome for authors and editors by dropping the “for England, Wales, Northern Ireland and Isle of Man”. In keeping with this we have removed it from the new suggested title. If you would rather, we put it back in then we can do that.

**Please avoid words such as "effect", implying causal inference, throughout the text unless supported by evidence.**

Thank you, we have been through the manuscript and swapped any mention of “effect” for “association between” or similar.

“We used Flexible Parametric Survival Models as described by Royston and Parmar to investigate the association between BMI category and the risk of revision.”

“Table 4. Hazard ratio (HR), 95% CI and p-value for coefficients of BMI categories extracted from the flexible parametric models to investigate the association of BMI with revision after primary TKR. Adjusted model adjusts for age, sex, ASA grade, indication for operation, year of primary TKR and fixation type. Both models were fitted on the hazard scale with 4 degrees of freedom.”

“Table 5. Hazard ratio (HR), 95% CI and p-value for coefficients of BMI categories extracted by Cox Proportional Hazards models to investigate the association of BMI with mortality within 90 days of primary TKR. Adjusted model adjusts for age, sex, ASA grade, indication for operation and year of primary TKR.”

**In the abstract, to "After adjustment ...", please add a summary of what has been adjusted for.**

Thank you, we have made this change

“After adjustment for age, sex, ASA grade, indication for operation, year of primary TKR and fixation type, patients with high BMI were more likely to undergo revision surgery within 10-years compared to those with “normal” BMI (Obese Class II HR 1.21 95%CI:1.10,1.32, Class III 1.13 95%CI:1.02,1.26).”

**In the abstract and main text, please quote p values alongside 95% CI, where available.**

This correction has been made. I have not included all instances in this document as there were so many.

**Please attach the analysis plan as a supplementary document, referred to in your Methods section.**

We do not have a formal analysis plan to publish.

**We did not find the RECORD checklist attached - please include this with your revision.**

Thank you for highlighting this, it must have been missed off with the revisions submission.

**In the checklist, please ensure that individual items are referred to by section (e.g., "Methods") and paragraph number, not by line or page numbers as these generally change in the event of publication.**

Thank you, we have made sure the checklist has been edited in this way.

**Please remove the "Role of the funding source" section from the Methods.**

We have now removed this.

**Please remove information on funding and competing interests from the end of the main text. In the event of publication, this information will appear in the article metadata, via entries in the submission form.**

This has now been removed leaving only the acknowledgements

## “Acknowledgements

We thank the patients and staff of all the hospitals who have contributed data to the National Joint Registry. We are grateful to the Healthcare Quality Improvement Partnership (HQIP), the National Joint Registry Steering Committee (NJRSC), and staff at the NJR Centre for facilitating this work. The views expressed represent those of the authors and do not necessarily reflect those of the NHS, the National Institute for Health Research, the Department of Health, NJRSC or HQIP who do not vouch for how the information is presented.**”**

**Throughout the article, please quote exact p values or, for smaller values, p<0.001.**

Thank you, we have made this change as suggested throughout the manuscript.

**Throughout the text, please style reference call-outs as follows: " ... most patients [1,2]." (noting the space preceding the square bracket and the absence of spaces within the brackets).**

We have made these changes in keeping with your advice.

**Please restrict "world's largest joint registry" to one mention, and add "to our knowledge", or similar.**

We have limited this to one entry in the abstract and one in the main text and removed other references. We have also changed the wording as suggested.

Abstract

“We investigated the associations between BMI and revision surgery, mortality and pain/function using what we believe to be the world’s largest joint replacement registry.”

Discussion

“We used what we believe is the largest joint replacement registry in the world with near complete coverage of all operations performed in the target population.”

**In the reference list, please use journal name abbreviations (e.g., "Lancet") consistently.**

Thank you, we have noted these errors and corrected them as you advise.

**Comments from Reviewers:**

***** Reviewer #1:**

**The authors have addressed my concerns and I now recommend publication**

Thank you very much for your support

**Peter Flom**

***** Reviewer #2:**

**The authors have done thorough work in revising the manuscript according to my and other reviewers' comments. Most importantly, the manuscript is much easier to follow now.**

**There are just a couple of issues that I'd suggest considering.**

**1) Supplementary data (S2) now shows number of cases that are still under follow-up at different timepoints. I think this is important information and do not see a reason to "hide" it in the supplementary data. I'd suggest adding the number of cases under follow-up into Figure 2. I may not be necessary to show the numbers for each BMI category. Just the total number could be enough.**

We agree that the number of joints at risk at each time point is useful information. The reason it had not been included is that Fig 2 is based on a model rather than S2 which is crude data. We have however included a caption that could be included below Fig 2 with this information in.

**“Numbers of knee replacements at risk at specified time points in the dataset from which the model was built.**

|  | Years since primary operation | | | | | | | |
| --- | --- | --- | --- | --- | --- | --- | --- | --- |
|  | 0 | 3 | 5 | 7 | 8 | 9 | 10 | 11 |
| Underweight | 1,338 | 807 | 505 | 236 | 153 | 66 | 20 | 0 |
| Normal | 49,860 | 28,400 | 17,064 | 8,395 | 8,395 | 2,085 | 199 | 36 |
| Overweight | 168,947 | 95,567 | 57,276 | 27,243 | 27,243 | 6,029 | 2,275 | 115 |
| Obese class I | 159,056 | 88,937 | 52,401 | 24,622 | 24,622 | 5,019 | 1,878 | 106 |
| Obese class II | 80,166 | 43,631 | 25,254 | 11,343 | 11,343 | 2,157 | 758 | 52 |
| Obese class III | 34,3433 | 18,672 | 10,728 | 4,752 | 4,752 | 917 | 336 | 21 |

**”**

**2) I think it would be important to show mortality during the follow-up years. In the current analyses regarding prosthesis survival, closure of follow-up and death are dealt with similarly (censoring of the case). As I already commented, mortality is likely different in different BMI groups (and similarly the type of ending the follow-up: closure of follow-up, revision, death) in long-term follow-up and from the viewpoint of considering risks and benefits of surgery, such differences are clinically relevant.**

Thank you for further clarifying this. We can understand your viewpoint that the competing risk of death may vary in different groups and have an impact on results. To reassure you, we have run a competing risks model (with death as a competing risk) by way of a sensitivity analysis (presented below) and the results are very similar to those presented from our original model where death is censored (treating every patient as though they are immortal). In view of this, we would rather remain with our original analyses previous presented.

**Table. Sub-hazard ratio (SHR), 95% CI and p-value for coefficients of BMI categories from a competing risk Fine and Gray proportional sub-hazards model to investigate the association of BMI with revision after primary TKR. Adjusted model adjusts for age, sex, ASA grade, indication for operation, year of primary TKR and fixation type.**

|  | **Unadjusted Model** | | | **Adjusted Model** | | |
| --- | --- | --- | --- | --- | --- | --- |
|  | **HR** | **95% CI** | **p-value** | **SHR** | **95% CI** | **p-value** |
| <18.5 kg/m^2^ | 0.93 | (0.58, 1.49) | 0.768 | 0.86 | (0.54, 1.37) | 0.530 |
| 18.5–24.99 kg/m^2^ (reference) | 1.00 |  |  | 1.00 |  |  |
| 25–29.99 kg/m^2^ | 1.15 | (1.05, 1.25) | 0.002 | 1.06 | (0.97, 1.16) | 0.170 |
| 30–34.99 kg/m^2^ | 1.29 | (1.19, 1.40) | <0.001 | 1.09 | (1.00, 1.19) | 0.045 |
| 35–39.99 kg/m^2^ | 1.58 | (1.45, 1.73) | <0.001 | 1.22 | (1.11, 1.34) | <0.001 |
| ≥40 kg/m^2^ | 1.69 | (1.52, 1.87) | <0.001 | 1.14 | (1.02, 1.27) | 0.019 |

We include a copy of the table where death is not treated as a competing risk used in the manuscript below for comparison.

**Table 4. Hazard ratio (HR), 95% CI and p-value for coefficients of BMI categories extracted from the flexible parametric models to investigate the association of BMI with revision after primary TKR. Adjusted model adjusts for age, sex, ASA grade, indication for operation, year of primary TKR and fixation type. Both models were fitted on the hazard scale with 4 degrees of freedom.**

|  | **Unadjusted Model** | | | **Adjusted Model** | | |
| --- | --- | --- | --- | --- | --- | --- |
|  | **HR** | **95% CI** | **p-value** | **HR** | **95% CI** | **p-value** |
| <18.5 kg/m^2^ | 0.96 | (0.60, 1.54) | 0.872 | 0.88 | (0.55, 1.41) | 0.608 |
| 18.5–24.99 kg/m^2^ (reference) | 1.00 |  |  | 1.00 |  |  |
| 25–29.99 kg/m^2^ | 1.12 | (1.03, 1.22) | 0.007 | 1.05 | (0.97, 1.14) | 0.252 |
| 30–34.99 kg/m^2^ | 1.26 | (1.16, 1.37) | <0.001 | 1.08 | (0.99, 1.18) | 0.073 |
| 35–39.99 kg/m^2^ | 1.54 | (1.41, 1.68) | <0.001 | 1.21 | (1.10, 1.32) | <0.001 |
| ≥40 kg/m^2^ | 1.64 | (1.48, 1.82) | <0.001 | 1.13 | (1.02, 1.26) | 0.026 |

**3) Figure 5 shows linear association between preoperative and postoperative OKS scores. The authors have only partly answered to my previous comment. The parts that remained unanswered are: "In its current form its value is questionable and interpretation is difficult: I think it is not that certain that association is linear but instead from lower scores the improvement might be greater." Secondly, there is possibly variation in individual patients' responses and one might hypothesize that variation might be greater in the obese groups. I'd suggest replacing Figure 3 with information based on actual observations (change from pre to postop in different BMI categories and its variation)." I'd like to emphasize that also variation might be of interest. In it's current form, I do not see much value for the current Figure 5.**

You are correct that the relationship could appear linear in this figure but this is not the aim of the figure. To reassure you, we have created a graph based on observed data (see below). The changes in Oxford Knee Score in all BMI categories

**Figure. Observed mean values of Oxford Hip Score with inter-quartile ranges stratified by BMI category.**

These observed data can already be seen in Table 3 of the manuscript and we discuss that the crude increase in OKS between pre- and 6-month post-operative assessments was similar across all BMI groups (range 18-20 points) and well above the minimal important change of 4/48 reported by Beard and colleagues. The variation in each category can also be seen in Table 3, where we present the inter-quartile ranges, demonstrating there is no apparent clinically significant difference in change in OKS between BMI categories.

Figure 3 was created as reassurance for a reviewer regarding the use of BMI as a categorial variable to demonstrate the relationship when BMI is treated as a continuous variable.
